# Out-of-hospital cardiac arrest survival in drug-related versus cardiac causes in Ontario: A retrospective cohort study

**DOI:** 10.1371/journal.pone.0176441

**Published:** 2017-04-26

**Authors:** Aaron M. Orkin, Chun Zhan, Jason E. Buick, Ian R. Drennan, Michelle Klaiman, Pamela Leece, Laurie J. Morrison

**Affiliations:** 1 Dalla Lana School of Public Health, University of Toronto, Toronto, Ontario, Canada; 2 Rescu, Li Ka Shing Knowledge Institute, St. Michael’s Hospital, University of Toronto, Toronto, Ontario, Canada; 3 Schwartz/Reisman Emergency Medicine Institute, Toronto, Ontario, Canada; 4 Institute of Health Policy, Management and Evaluation, University of Toronto, Toronto, Ontario, Canada; 5 Department of Family and Community Medicine, University of Toronto, Toronto, Ontario, Canada; 6 Institute of Medical Science, Faculty of Medicine, University of Toronto, Toronto, Ontario, Canada; 7 Division of Emergency Medicine, St. Michael’s Hospital, University of Toronto, Toronto, Ontario, Canada; 8 Public Health Ontario, Toronto, Ontario, Canada; Osaka University Graduate School of Medicine, JAPAN

## Abstract

**Background:**

Drug overdose causes approximately 183,000 deaths worldwide annually and 50,000 deaths in Canada and the United States combined. Drug-related deaths are concentrated among young people, leading to a substantial burden of disease and loss of potential life years. Understanding the epidemiology, patterns of care, and prognosis of drug-related prehospital emergencies may lead to improved outcomes.

**Methods:**

We conducted a retrospective cohort study of out-of-hospital cardiac arrests with drug-related and presumed cardiac causes between 2007 and 2013 using the Toronto Regional RescuNet Epistry database. The primary outcome was survival to hospital discharge. We computed standardized case fatality rates, and odds ratios of survival to hospital discharge for cardiac arrests with drug-related versus presumed cardiac causes, adjusting for confounders using logistic regression.

**Results:**

The analysis involved 21,497 cardiac arrests, including 378 (1.8%) drug-related and 21,119 (98.2%) presumed cardiac. Compared with the presumed cardiac group, drug-related arrest patients were younger and less likely to receive bystander resuscitation, have initial shockable cardiac rhythms, or be transported to hospital. There were no significant differences in emergency medical service response times, return of spontaneous circulation, or survival to discharge. Standardized case fatality rates confirmed that these effects were not due to age or sex differences. Adjusting for known predictors of survival, drug-related cardiac arrest was associated with increased odds of survival to hospital discharge (OR1.44, 95%CI 1.15–1.81).

**Interpretation:**

In out-of-hospital cardiac arrest, patients with drug-related causes are less likely than those with presumed cardiac causes to receive bystander resuscitation or have an initial shockable rhythm, but are more likely to survive after accounting for predictors of survival. The demographics and outcomes among drug-related cardiac arrest patients offers unique opportunities for prehospital intervention.

## Introduction

Drug overdose causes approximately 50,000 deaths in Canada and the United States combined, and 183,000 deaths worldwide annually.[1,2,3] In the United States, United Kingdom, and Australia, overdose is responsible for more deaths each year than motor vehicle collisions[4,5,6]. Drug-related deaths are concentrated among young people, leading to a substantial burden of disease. In Ontario, years of life lost due to opioid-related mortality nearly tripled between 1992 and 2010, from 1.3 to 3.3 years per 1,000 population.[7] From 2014 to 2015, British Columbia overdose death rates rose by over 30% to 10.2 deaths per 100,000, prompting the declaration of a provincial public health emergency in April 2016.[8,9] In response to rising opioid fatality rates, in March 2016 Health Canada amended the prescription drug list to allow the non-prescription use of naloxone for opioid overdose reversal.[10].

Cardiac arrest is the common clinical endpoint in all drug-related fatalities, but the epidemiology, prehospital management, and prognosis of patients with drug-related out-of-hospital cardiac arrest has been historically understudied. North American and European studies show that cardiac arrest in opioid overdose carries a poor prognosis—between 0% and 12.7% survive to hospital discharge.[11,12,13,14,15] There are no published studies on the characteristics and outcomes of drug-related cardiac arrest specific to the Canadian context. Canadian overdose surveillance and data systems are severely underdeveloped.[16,17,18] Canadian studies using Coroner’s data, death registries, and chart reviews provide limited information about the clinical circumstances of drug-related fatalities.[19,20,21].

Understanding the epidemiology, patterns of care, and prognosis of drug-related prehospital emergencies may lead to improved outcomes. The objectives of this study were to compare the epidemiology, resuscitative indicators, and outcomes of out-of-hospital cardiac arrests attributed on-scene to a drug-related cause versus those with a presumed cardiac aetiology in Ontario. Specifically, we sought to determine if drug-related out-of-hospital cardiac arrest is independently associated with survival.

## Methods

### Data source and study type

This was a population-based retrospective cohort study of survival in out-of-hospital cardiac arrest with drug-related versus presumed cardiac causes, using data from Rescu Epistry, the Toronto Regional RescuNET cardiac arrest database.

Rescu Epistry is comprised of data points from the Resuscitation Outcomes Consortium (ROC) Epistry-Cardiac Arrest database and the Strategies for Post Arrest Resuscitation Care database. Rescu Epistry database methodologies are described elsewhere. [22,23].

Rescu Epistry incorporates data from a network of seven paramedic services (Toronto, York, Peel, Durham, Halton, Simcoe, Muskoka), local fire departments, the provincial air ambulance service, and 44 participating destination hospitals. Rescu Epistry variables include patient characteristics and demographics, dispatch and call characteristics, geographic data, prehospital and in-hospital interventions and outcome variables.[24] Data abstractors collect data from standardized prehospital call reports, emergency department (ED) and in-hospital records, and validate data through a quality assurance program.[17] CPR quality data such as chest compression rate, depth, and compression fraction (proportion of resuscitative period during which chest compressions are performed) are downloaded directly from defibrillator software into the Rescu Epistry database when available.

The Rescu Epistry protocol is approved with waiver of individual patient consent by the institutional research ethics boards of each destination hospital and participating service. Data was accessed anonymously. The study was designed and reported according to STROBE standards for cohort studies.[25].

### Population and setting

The study population included all paramedic-treated cardiac arrest patients receiving chest compressions or a defibrillator shock between January 1, 2007 and December 31, 2013 across the Rescu Epistry regions in southern Ontario.

Approximately 10,000 paramedics and first responders provide tiered response for cardiac arrests within the catchment regions. These regions encompass urban, suburban and rural areas, and contained a growing population of 6.6 million people over the study period (51% of the Ontario population). Fire department personnel and primary care paramedics are dispatched to calls for cardiac arrests to provide basic life support. Advanced Care paramedics are dispatched when available, and are trained to administer advanced life support medication and airway interventions.

### Case definition and exclusions

**Fig 1:**
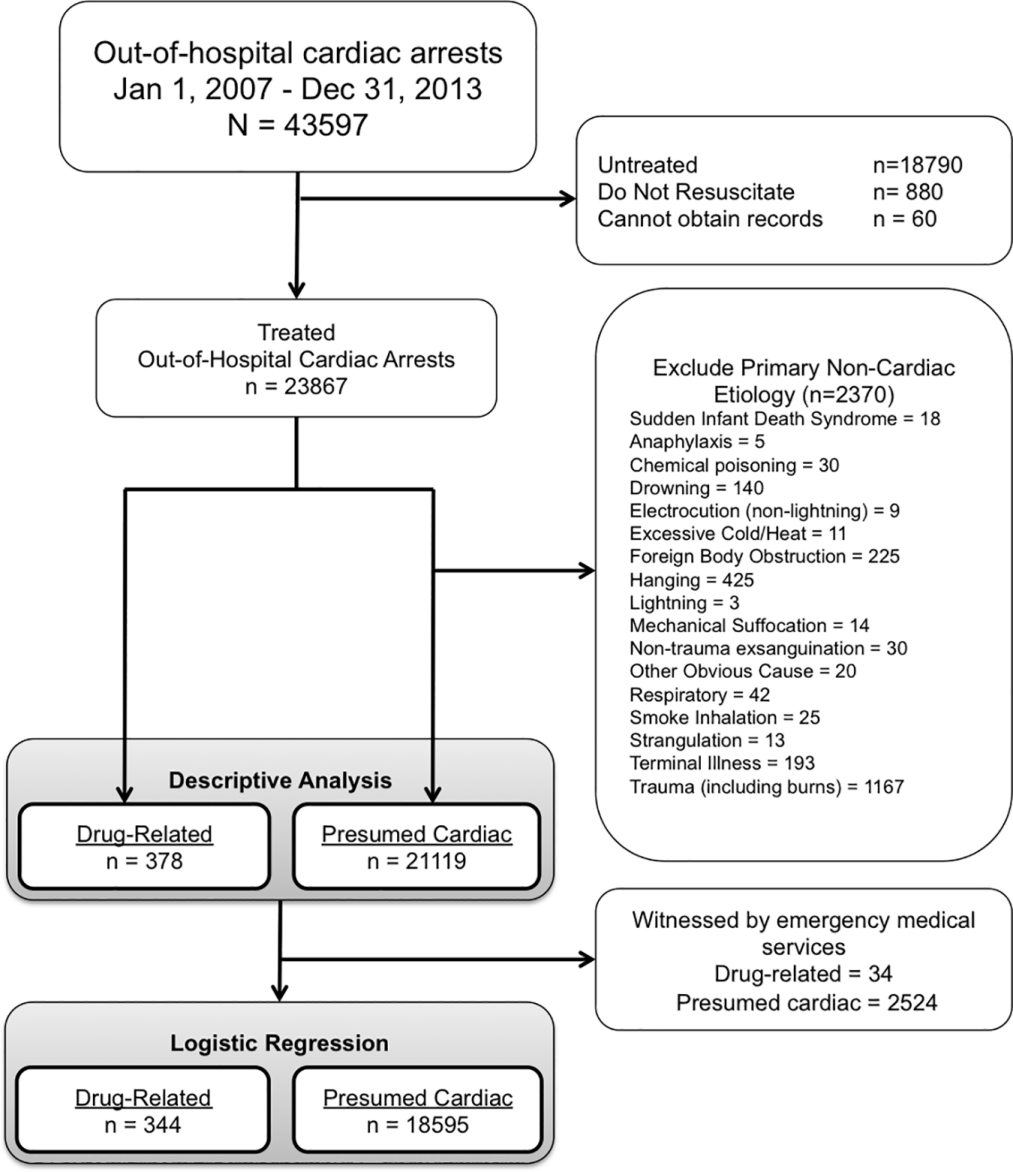
Flow diagram of patient inclusions and exclusions.

Drug-related out-of-hospital cardiac arrest (“drug-related cardiac arrest” hereafter) was defined as an out-of-hospital cardiac arrest where data abstractors identified drugs as the main aetiology of cardiac arrest or a contributing factor in the cardiac arrest from paramedic and in-hospital documentation. This generally involved written descriptions of the context of the cardiac arrest, such as a patient who collapses and there are empty pill bottles or a tourniquet on the patient’s arm.[22] Although specific drugs were not documented, they included prescribed and over-the-counter medications, illicit substances and alcohol. This excludes all cases of chemical poisoning (carbon monoxide, methanol etc.). This includes both intentional and unintentional drug overdose. The full case definition is provided as S1 Appendix.[22] As with other comparative cardiac arrest studies, out-of-hospital cardiac arrest with a presumed cardiac cause was defined as all out-of-hospital cardiac arrest cases with no other obvious non-cardiac cause (Fig 1).[26,27].

Cardiac arrest patients with a pre-existing directive to withhold cardiopulmonary resuscitation (Do Not Resuscitate, DNR) were excluded from the analysis. Patients who met standards for obvious death were also excluded.[28] Cases of paramedic-witnessed arrest were excluded from the regression model to focus on variables affecting survival in patients in whom cardiac arrest occurred prior to paramedic arrival (Fig 1).

Following Ontario Medical Termination of Resuscitation protocols, paramedics may also terminate resuscitation in the prehospital setting. In these cases, the deceased patient would not be transported to hospital.[29].

### Outcomes

The primary outcome was survival to hospital discharge. We assessed survival with favourable neurological outcome, return of spontaneous circulation (ROSC) for ≥20 minutes after ED arrival, and admission to hospital as secondary outcomes. We defined favourable neurological outcome as score on the a Modified Rankin Scale (mRS) ≤ 2.[30].

### Analysis

Incidences were calculated using population counts from the Statistics Canada 2006 and 2011 censuses.[31] We computed case fatality rates as the quotient of deaths and cases for drug-related and cardiac controls respectively. We compared age- and sex-standardized case fatality rates using the direct method, with the 2011 Statistics Canada Ontario population as the standard.[32].

We conducted a descriptive comparison of cardiac arrest cases with drug-related and presumed cardiac causes based on patient characteristics, response and transport variables, prehospital resuscitation procedure, in-hospital and post-arrest procedures, and outcome variables. We compared the two groups on the basis of mean compression rate, mean compression depth, and compression fraction, defined as the proportion of time when the patient received chest compressions during the first ten minutes of resuscitation. Continuous variables approximated Gaussian distributions. Comparisons of continuous variables, discrete and categorical variables, and repeat measures were made using the Student’s *t-*test, χ^2^-test and non-parametric Wilcoxon rank sum test, respectively.

We built a logistic regression model to determine the relationship between drug-related status and survival to hospital discharge. In the absence of an established theoretical model to drive survival in drug-related cardiac arrest, we explored the role of all variables involved in the descriptive analysis as candidate variables following a stepwise backward elimination procedure.

We retained the following variables in the final regression model: age, initial cardiac rhythm (shockable vs. non-shockable rhythm), witnessed status, bystander CPR, bystander use of an automated external defibrillator (AED), pickup location (public settings vs. private residences), advanced versus basic responder, emergency medical services (EMS) response time, the use of epinephrine, and an advanced airway device. Use of epinephrine or advanced airway device variables were tested as effect modifiers on the advanced vs. basic life support covariate.

Emergency medical services response time was defined as the interval from when a 911 call was received until the arrival of a 911-initiated first responder, including firefighters and paramedics. EMS response times were missing for 1617 cases (7%) (23 drug-related and 1594 cardiac cases, 6.7% and 8.6%). We confirmed that 1423 (88%) of these missing values occur when a first responder service does not provide their data to Rescu Epistry. These values were determined to be statistically missing completely at random according to Little’s test. [33] We used multiple imputation to generate missing values and conducted a sensitivity analysis to determine if excluding these cases from the logistic regression would alter results meaningfully.

We conducted an *a priori* sensitivity analysis to determine if results would alter substantially if the definition of drug-related cases were expanded to include any arrest where the patient had a past history of illegal drug use as abstracted from paramedic or hospital records.

Analyses were conducted using SAS software v.9.4 (SAS Institute, Cary, NC) and Microsoft Excel v.14.4.5 (Microsoft Corporation, Seattle, WA).

## Results

There were 43,597 out-of-hospital cardiac arrest patients during the study period, of which 23,867 were treated by paramedics. 2,370 patients (9.9%) with a traumatic or other obvious cause were excluded to define cardiac arrest patients with a presumed cardiac cause. The final study population included 21,497 patients, 378 drug-related and 21,119 presumed cardiac (1.8% and 98.2% of all treated out-of-hospital arrests, respectively) (Fig 1).

### Incidence and adjusted case-fatality rates

The mean incidence of treated out-of-hospital cardiac arrest during the study period was 0.85 and 46.08 cases per 100,000 people per year for drug-related and presumed cardiac causes respectively (Table 1).

**Table 1:** Annual incidence of cardiac arrest, drug-related and presumed cardiac causes, 2007–2013.

Year	Total Population*	Drug-Related		Presumed Cardiac	
		Count	Incidence (Cases/100,000/yr)	Count	Incidence (Cases/100,000/yr)
2007	4,703,761	37	0.79	2117	45.01
2008	4,703,761	42	0.89	2336	49.66
2009	5,142,967	51	0.99	2522	49.04
2010	6,035,679	38	0.63	2816	46.66
2011	6,558,301	52	0.79	2779	42.37
2012	6,558,301	64	0.98	2877	43.87
2013	6,558,301	57	0.87	3014	45.96
Mean Incidence over Study Period			0.85		46.08

* Includes census population estimates of Toronto Regional RescuNET service regions; Durham, Halton, Muskoka, Peel, Simcoe, Toronto and York health regions. All population increases are attributed to population growth.

The crude case fatality rate was 912.8 per 1,000 cases for drug-related cardiac arrest and 930.7 per 1,000 cases for arrest of presumed cardiac cause. The direct age- and sex-adjusted case fatality rates were 935.7 per 1000 drug-related cases and 888.3 per 1000 presumed cardiac cases (S2 Appendix).

### Descriptive analysis

**Fig 2:**
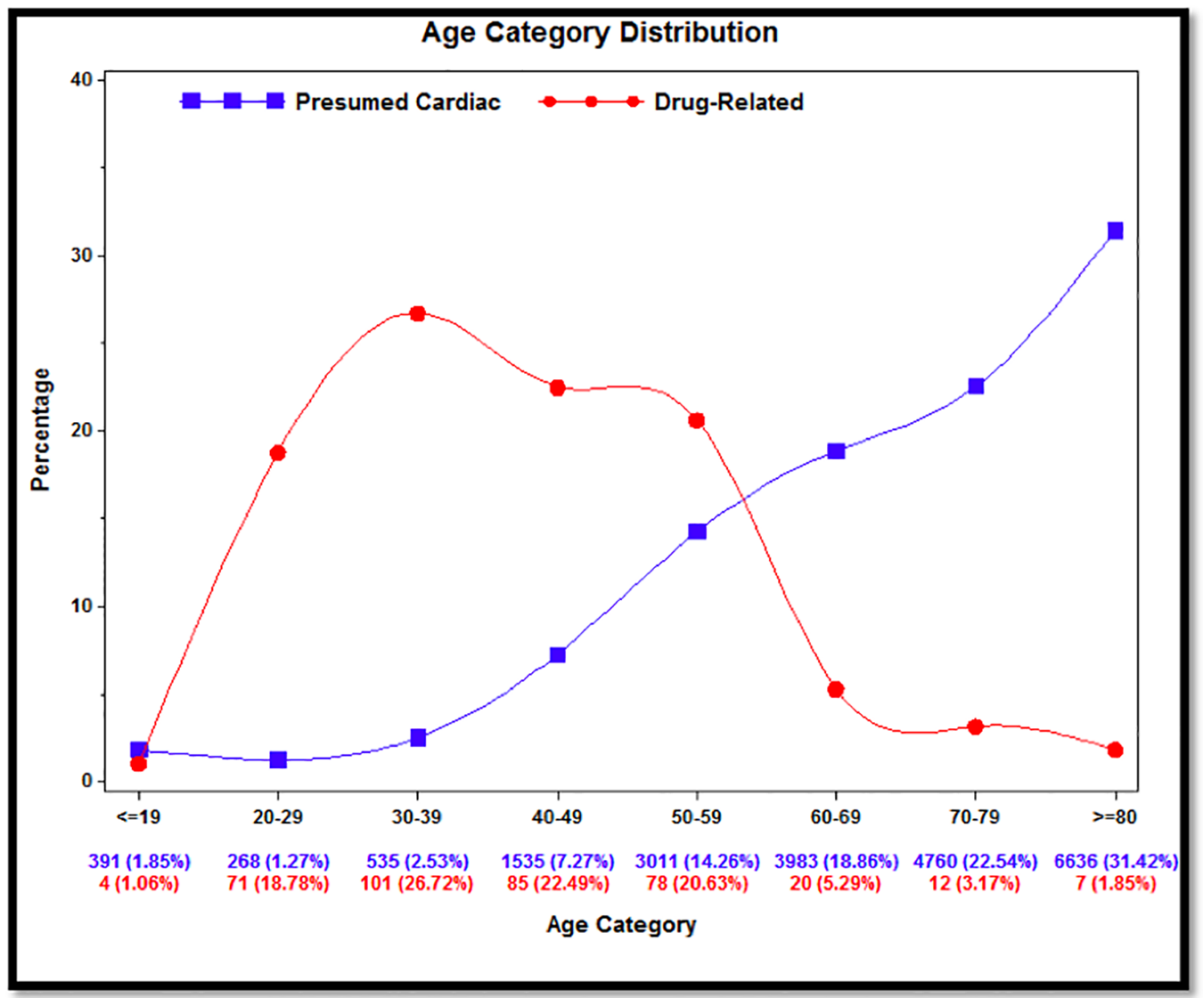
Cardiac arrest age distribution by group.

Drug-related cases and presumed cardiac cases differed demographically, and in terms of bystander response and transportation to the emergency department, prehospital interventions, and outcomes (Table 2). Drug-related cases were, on average, 25.9 years younger than cardiac comparators with different age distributions (Fig 2). The 50–59 age category represented the point of intersection in the frequency of the two groups, and was therefore used as the reference age in the subsequent logistic regression. There was no significant difference in sex or proportion of events occurring in public settings.

**Table 2:** Descriptive analysis—Cardiac arrest with drug-related and presumed cardiac causes.

	Drug-Related	Presumed Cardiac	p-value
**Patient Characteristics**			
Age Mean, yr[95% CI]	42.68 [41.19–44.18]	68.50 [68.26–68.74]	**<0.0001**
Female, n(%)	130/378 (34.39)	7477/21119 (35.40)♮	0.90
Public Location, n(%)	48/378 (12.70)	3299/21086 (15.65)	0.12
Past History–Drugs, n(%)	146/378 (38.62)	633/21119 (3.00)	**<0.0001**
**Response**			
Bystander Resuscitation*, n(%)	112/344 (32.6)	7480/18595 (40.2)	**0.004**
Witnessed (Bystander or EMS), n(%)	104/378 (27.51)	10888/21119 (51.56)	**<0.0001**
EMS Response Time*, minutes [95%CI](n)	6.17 [5.87–6.47] (321)	6.40 [6.35–6.45] (17001)	0.23
ALS Response, n(%)	357/378 (94.4)	19010/21119 (90.01)	**0.004**
Transported to ED, n(%)	185/378 (48.94)	11969/21119 (56.67)	**0.003**
**Prehospital Resuscitation Procedure**			
Shockable Cardiac Rhythm, n(%)**	28/360 (7.78)	4790/20537 (23.32)	**<0.0001**
Any prehospital Defibrillation, n(%)	76/378 (20.11)	7356/21119 (34.83)	**<0.0001**
Time of call to first shock ♮♮, minutes[95% CI]	13.17[10.55–15.80]	12.80 [12.58–13.02]	0.80
Epinephrine, n(%)	285/378 (75.40)	15273/21119 (72.32)	0.15
Amiodarone, n(%)	15/378 (3.97)	1948/21119 (9.22)	**0.0004**
Airway—Advanced, n(%)	301/378 (79.63)	15996/21119 (75.74)	0.10
Compressions/min, mean[95% CI]	108.6(106.6–110.6)	108.7(108.4–108.9)	0.46
Comp’n Depth, cm, mean [95% CI]	4.62 (4.45–4.78)	4.59 (4.57–4.61)	0.97
**Outcome Variables**			
Discharge from Hospital, n(%)	36/378 (9.52)	1761/21119 (8.34)	0.41
ROSC ≥20 minutes after ED arrival §§, n(%)	75/377 (19.89)	4543/21013 (21.62)	0.42
Admission to Hospital♮, n(%)	76/378 (20.11)	4192/21119 (19.85)	0.90
Favourable Neurological Outcome (MRS ≤2)§, n(%)	15/185 (8.11)	844/9936 (8.49)	0.81

ALS: Advanced Life Support; AED: Automated External Defibrillator; CPR: Cardiopulmonary resuscitation; MRS: Modified Rankin Score; EMS: Emergency Medical Services; ROSC: Return of Spontaneous Circulation; ED: Emergency Department

*EMS-witnessed events excluded

♮♮Among Shockable patients, n = 4344

§ for cases after Jan 1, 2011

**comparison made without missing or unknown values

§§ 106 missing values, 1 in the drug-related group

♮ 2 missing values.

Drug-related events were less often witnessed by a bystander, and were less likely to receive bystander CPR. Paramedics were less likely to find drug-related cases in ventricular fibrillation or ventricular tachycardia, or perform any defibrillation during the resuscitation. Drug-related cases were more likely to receive treatment from ALS-trained paramedics, and were more likely to have resuscitation terminated without transportation to hospital.

No significant differences were observed in unadjusted outcomes between drug-related and presumed cardiac cases. Both groups were discharged from hospital and had a favourable neurological outcome at roughly the same rate (Table 2).

Chest compression rate and fraction were available for approximately 65% of cases in both groups, and compression depth data was available for 55% of cases based on regional variation in defibrillator technology. There was no statistical difference between drug-related and presumed cardiac cases with respect to mean compression rate or depth.

**Fig 3:**
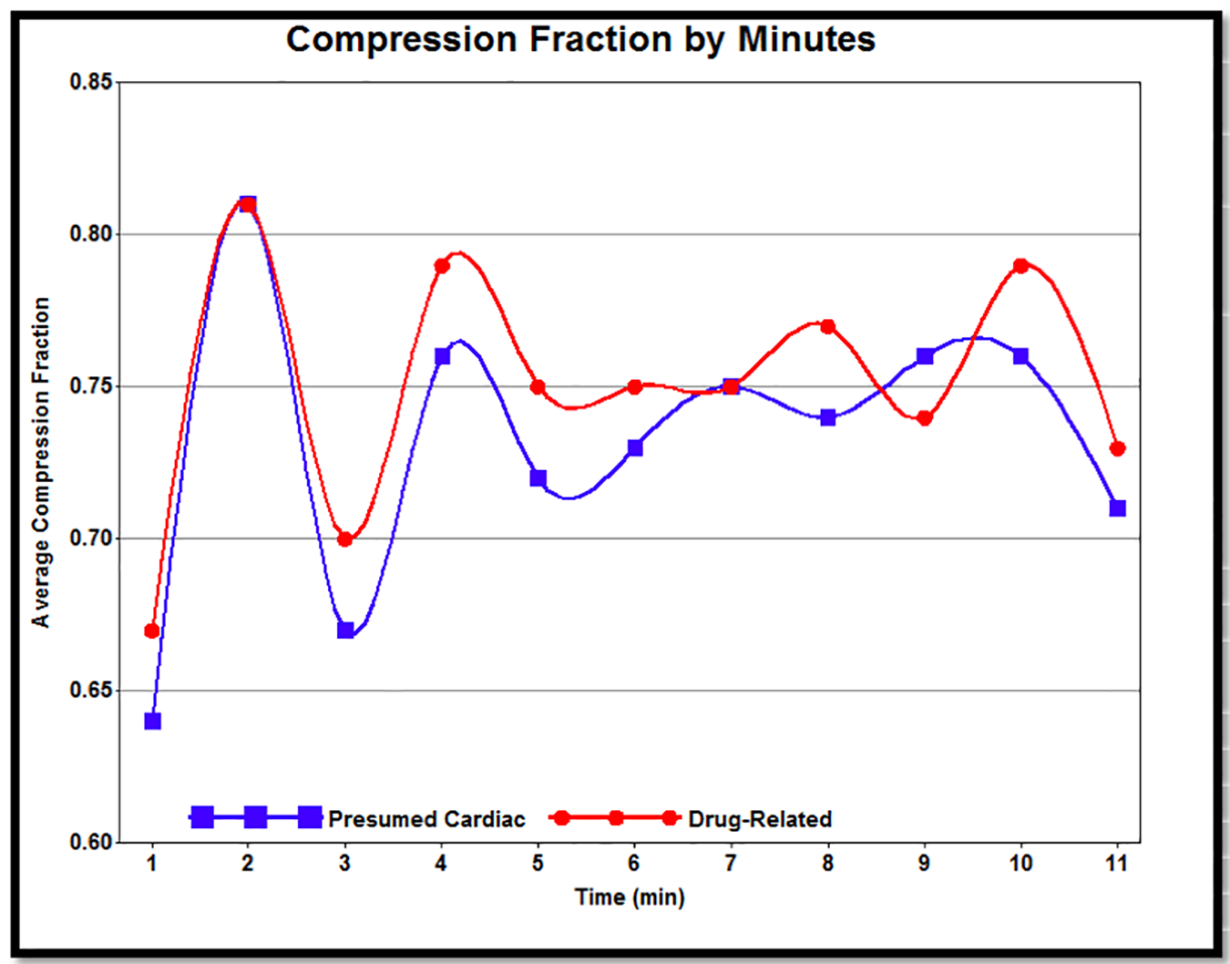
Smoothed plot of compression fraction by minute.

Fig 3 shows the mean compression fraction per minute for the first 10 minutes of resuscitation. Drug-related cardiac arrests received a higher proportion of chest compressions over time than their presumed cardiac counterparts (*p* = 0.02 over 10 minutes, *p =* 0.0016 over the first 5 minutes).

#### Logistic regression

Table 3 provides summary odds ratios and 95% confidence intervals for all variables retained in the backwards elimination procedure. When adjusted for other covariates, drug-related cases had significantly greater odds of survival compared to presumed cardiac patients (OR 1.44, 95%CI 1.15–1.81). Having an advanced care paramedic response, a bystander-witnessed arrest, an initial shockable cardiac rhythm, bystander defibrillator use or a cardiac arrest in a public location were also independently associated with a greater odds of survival.

**Table 3:** Logistic regression model–Effect of drug-related vs. presumed cardiac cause on survival to hospital discharge.

Variable/Covariate	OR	95% CI
Drug-Related vs. Presumed Cardiac (ref.)	1.44	1.15–1.81
Age		
≤19	1.32	0.93–1.89
20–29	2.15	1.48–3.12
30–39	2.07	1.57–2.73
40–49	1.09	0.89–1.33
50–59 (ref.)	-	-
60–69	0.86	0.74–1.01
70–79	0.60	0.51.0.71
≥80	0.30	0.25–0.36
EMS response time (per minute)	0.91	0.89–0.95
ALS response	2.65	2.33–3.02
Witnessed OHCA	1.78	1.64–1.93
Epinephrine given	0.31	0.28–0.34
Advanced airway	0.80	0.73–0.86
VF or VT	3.16	2.91–3.43
Bystander Resuscitation	1.11	1.04–1.20
Public vs. private location	1.27	1.18–1.37

Model fit statistics: R^2^ = 0.1745, Adjusted R^2^ = 0.4399, c = 0.905.

EMS: Emergency Medical Services, ALS: Advanced Life Support, OHCA: Out-of-hospital cardiac arrest, VF: Ventricular Fibrillation, VT: Ventricular Tachycardia.

Our sensitivity analyses revealed that the observed effects remained robust when cases with imputed values were removed or when the drug-related case definition was expanded to include all patients with a past history of drug use (S3 Appendix).

## Interpretation

In this study, drug-related cardiac arrest patients had similar unadjusted rates of survival to hospital discharge and favourable neurological status compared to presumed cardiac patients. However, drug-related cardiac arrests were less frequently witnessed by a bystander, less likely to be found with an initial shockable cardiac rhythm, and less likely to receive prehospital defibrillation than presumed cardiac cases. These differences are generally associated with diminished odds of survival.[34] This incongruity between rates of survival in drug-related cardiac arrest and other variables typically associated with survival is the most notable finding of our study. Drug-related cardiac arrest patients were, on average, younger than their cardiac counterparts, although the similarity in crude survival rates for both groups was not due to age differences (Fig 2). Our standardized case fatality rate calculations confirmed that drug-related cardiac arrest carried a higher age- and sex-standardized risk of death than cardiac arrest with a presumed cardiac cause.

Using a population-based registry of clinical records, our study delivers the first Canadian estimate of the incidence of treated drug-related out-of-hospital cardiac arrest and case-fatality rates. Emerging cardiac arrest registries and linkage with administrative and Coroner’s data could contribute to much-needed overdose surveillance systems.[17,18].

Our findings align with other studies showing that opioid-related cardiac arrests affect younger populations and have a significantly lower rate of initial shockable cardiac rhythm than patients with non-drug-related causes, yet have a similar or greater chance of survival independent of age. [11,12,13,35] When compared to the 2014 study by Koller *et al* and the 2016 study by Salcido *et al*, we found lower rates of survival among drug-related cardiac arrest patients (9.5% vs. 18.9% and 12.7% respectively).[11,12] In comparison with our findings, patient’s with opioid-related cardiac arrest in the Koller study had a much higher rate of witnessed arrests (48.4% vs. 27.5%) and initial shockable cardiac rhythms (15.6% vs. 7.78%). This may account for the higher rates of survival reported by Koller *et al*. In the subset Canadian settings in the Salcido study, survival rates were similar to our own. Though our study findings align with the Salcido study’s pooled data from heterogeneous settings in Canada and the United States, our study offer the first specific analysis of drug-related cardiac arrest in the unique Canadian geographical, drug policy, and healthcare context.[11,36].

We observed higher odds of survival among drug-related cases in the multivariate logistic regression. These observations may be driven by unmeasured differences between the drug-related and presumed cardiac groups. Candidate factors include a differential effectiveness of clinical interventions such as airway management and naloxone administration, reversible underlying physiological processes in drug-related cardiac arrest, or other variables such as comorbidities independent of age.

Canadian studies demonstrate that drug-related deaths account for more years of life lost than alcohol use disorders, pneumonia, HIV/AIDS, or influenza.[7] Our findings reveal disparities in the bystander and professional care received by cardiac arrest patients with a drug-related versus presumed cardiac cause. Given that drug-related cardiac arrest patients receive fewer of the interventions generally associated with survival, reducing drug-related deaths by enhancing professional and bystander care could deliver population health effects.

Prehospital resuscitation guidelines for drug-related emergencies do not differ substantially from standard resuscitation guidelines.[37] Healthcare providers perceive drug-related cardiac arrest treatment, outcomes, and patient characteristics differently to other causes of cardiac arrest, which may bias practices and treatment decisions.[38] Future research could identify cause-specific out-of-hospital cardiac arrest protocols that would enhance care in this subpopulation and refine prehospital providers’ awareness and approach to drug-related emergencies. Deaths may be averted by enhancing bystanders’ abilities to prevent and recognize overdose, activate 911 services, and administer effective first aid interventions such as bystander naloxone administration and chest compressions.[37].

### Limitations

Our case definition and data set does not differentiate illicit drugs, alcohol, and prescription medications. American surveillance data demonstrates that less than 20% of drug-related deaths are associated primarily with alcohol, cocaine, or psychostimulants.[39,40] Most drug-related cases in our study were likely due to opioids.

Other studies have defined overdose-associated cardiac arrest based on naloxone administration, toxicology screens, or explicit indication of acute drug use at the time of the cardiac arrest.[11,12,13] This approach may lack sensitivity because resuscitation guidelines during the study period did not recommend naloxone administration in cardiac arrest.[12,15,41] Detailed data collection in the Rescu Epistry permits a more valid and reliable definition of drug-related cardiac arrest, based on first responders’ clinical and on-scene observations. This approach may be subject to observer biases, but offers a superior pragmatic case definition in comparison with subsequent toxicological tests or antidote administration records. First responders’ immediate assessments of drug-related aetiology must guide prehospital and in-hospital resuscitative interventions for this population.

Our analysis focused on the variables and information immediately available to guide prehospital resuscitation decisions, and therefore did not adjust for medications or comorbidities. These could represent unmeasured confounders. With adjustment for age, medications and comorbidities do not consistently predict out-of-hospital cardiac arrest survival and are not routinely included in comparative analyses of cardiac arrest outcomes.[42] We adjusted for age using both regression and standardization tables.

Misclassification or exclusions may occur for unresponsive patients not in cardiac arrest, or secondary trauma cases following cardiac arrest. The retrospective nature of our study limits causal inferences, especially because the mechanisms and physiology of drug-related cardiac arrest are not fully understood. Our study took place in a mostly urban and suburban population with a robust first response system and may not be generalizable to contexts with less developed prehospital care.

## Conclusion

Out-of-hospital cardiac arrest with a drug-related cause carries a similar chance of survival as cardiac arrest with a presumed cardiac cause. However, these two groups differ substantially in terms of their demographic characteristics, circumstances of cardiac arrest, and prehospital clinical course. With adjustment for these factors, patients with drug-related cardiac arrest had greater odds of survival than cardiac arrest with a presumed cardiac cause. The lower rates of bystander resuscitation and transport to hospital in drug-related cardiac arrest point to a need for initiatives that increase the frequency of bystander interventions and refine out-of-hospital resuscitation protocols for this group.

## Supporting information

S1 Appendix Drug-related out-of-hospital cardiac arrest case definition. (DOCX)

S2 Appendix Direct age and sex standardization table–Cardiac arrest, drug-related and presumed cardiac causes. (DOCX)

S3 Appendix Sensitivity analysis of drug-related cardiac arrest case definition. (DOCX)

S4 Appendix STROBE statement checklist. (DOC)
