# Supplementary material for: Out-of-hospital cardiac arrest survival in drug-related versus cardiac causes in Ontario: A retrospective cohort study
Source: PLoS One. 2017 Apr 26;12(4):e0176441. doi: 10.1371/journal.pone.0176441 (PMC5405992; doi:10.1371/journal.pone.0176441)
Supplement: S1 Appendix — (DOCX) [file pone.0176441.s001.docx]

***Appendix 1: Drug-Related Out-of-Hospital Cardiac Arrest Case Definition***

Our case definition includes the following two codings from the Rescu Epistry database. These coding definitions are taken verbatim from the Rescu Epistry data elements, definitions and reference codes. <**Error! Bookmark not defined.**>

1. *Etiology of arrest—site classification:* Indicate the apparent cause of arrest, either 'obvious' or 'no obvious', based only on information documented in the pre-hospital care record. It is anticipated that the majority of arrests will fall into the 'no obvious cause' category and will include those cases that are presumed cardiac (NEMSIS 2250) or do not clearly fit in any of the 'obvious cause' categories listed and defined below. The selected site classification for etiology of arrest may differ from that documented for the 'field classification.' The field classification will have been documented based upon local provider protocol, definitions, and/or habit. The site classification will be based upon interpretation of the complete pre-hospital record and conformance to the below definitions. Mark 'obvious cause' only when the cause of out of hospital cardiac arrest clearly meets the defined criteria. The Epistry PIs expect that 'obvious cause' may be rarely selected in the data set. Arrest patients with an 'obvious cause' become a unique subgroup that may have different treatments and outcomes. Definitions of obvious causes follow: […]

Drug poisoning (intentional or unintentional, includes ethanol): This category includes prescribed medications, recreational drugs, and ethanol. Intentional drug overdose may include cases where ingestion of a drug (i.e., prescribed or over the counter medication, recreational drugs including alcohol) for the purposes of suicide is clear (suicide note, witnesses confirm discussion of suicidal intent or witnessed clear impulsive intentional ingestion temporarily related to the collapse). Unintentional drug overdose may include cases where witnessed inhaled or intravenous or oral recreational drug use immediately precedes the collapse. The Drug Poisoning category includes cases where witnesses confirm the situation, for example an injection of heroin just prior to collapse or where the evidence strongly suggests immediate use prior to arrest (e.g., tourniquet on arm and empty syringe at side). (may include drug poisoning NEMSIS 9530)

Examples of cases that fit this definition; a victim collapses and there are empty pill bottles or ethanol containers at the scene without clear evidence of suicidal intent.

Examples of cases that DO NOT fit this definition; patient with a history of recreational drug use within 24 hours of the event (e.g., adolescent found collapsed at a party or the known alcoholic found dead the morning after heavily imbibing); classify these types of cases as 'no obvious cause' (presumed cardiac).

2. Contributing factors: This section is intended to capture the variety of factors that were observed by, or reported to, the EMS responders during the course of prehospital care. The site coordinator will mark all applicable conditions that are documented in the PCR (whether narrative or check item format) that may have been related to the cardiac arrest. It is not necessary for a 'contributing factor' to meet the same burden of proof as does 'obvious cause' for Etiology of Arrest: Site Classification. Check all contributing factors that appear to be directly related to this cardiac arrest. Definitions of contributing factors follow: […]

Drug poisoning (intentional or unintentional, includes ethanol): Includes prescribed or over the counter medications, recreational drugs, and ethanol (alcohol). May include drug poisoning NEMSIS 9530).
