# Supplementary material for: Out-of-hospital cardiac arrest survival in drug-related versus cardiac causes in Ontario: A retrospective cohort study
Source: PLoS One. 2017 Apr 26;12(4):e0176441. doi: 10.1371/journal.pone.0176441 (PMC5405992; doi:10.1371/journal.pone.0176441)
Supplement: S2 Appendix — (DOCX) [file pone.0176441.s002.docx]

***Appendix 2: Direct Age and Sex Standardization Table – Cardiac Arrest, Drug-Related and Presumed Cardiac Causes***

| Drug-Related |  | Age Categories | **Deaths** | Pop | | Age-specific death rate/1000 | | Presumed Cardiac | Deaths | Pop | Age-specific death rate/1000 | | Adjusted Rates | Ontario Ref. Population | Expected Deaths DROHCA | Expected Deaths COHCA | |
| --- | --- | --- | --- | --- | --- | --- | --- | --- | --- | --- | --- | --- | --- | --- | --- | --- | --- |
|  | M | <=19 | 1 | 1 | | 1000 | |  | 195 | 219 | 890.4109589 | |  | 1561190 | 1561190 | 1390100.685 | |
|  |  | 20-29 | 41 | 43 | | 953.4883721 | |  | 137 | 163 | 840.4907975 | |  | 1615930 | 1540770.465 | 1358174.294 | |
|  |  | 30-39 | 49 | 58 | | 844.8275862 | |  | 275 | 319 | 862.0689655 | |  | 1642960 | 1388017.931 | 1416344.828 | |
|  |  | 40-49 | 46 | 50 | | 920 | |  | 825 | 930 | 887.0967742 | |  | 1975495 | 1817455.4 | 1752455.242 | |
|  |  | 50-59 | 44 | 48 | | 916.6666667 | |  | 1689 | 1939 | 871.0675606 | |  | 2065735 | 1893590.417 | 1799394.747 | |
|  |  | 60-69 | 14 | 14 | | 1000 | |  | 2249 | 2526 | 890.3404592 | |  | 1279805 | 1279805 | 1139462.171 | |
|  |  | 70-79 | 6 | 6 | | 1000 | |  | 2665 | 2839 | 938.7108137 | |  | 367690 | 367690 | 345154.5791 | |
|  |  | >=80 | 4 | 4 | | 1000 | |  | 3125 | 3204 | 975.3433208 | |  | 194540 | 194540 | 189743.2896 | |
|  | F | <=19 | 2 | 2 | | 1000 | |  | 130 | 145 | 896.5517241 | |  | 1483215 | 1483215 | 1329778.966 | |
|  |  | 20-29 | 21 | 24 | | 875 | |  | 67 | 79 | 848.1012658 | |  | 1667585 | 1459136.875 | 1414280.949 | |
|  |  | 30-39 | 31 | 35 | | 885.7142857 | |  | 126 | 145 | 868.9655172 | |  | 1770150 | 1567847.143 | 1538199.31 | |
|  |  | 40-49 | 24 | 27 | | 888.8888889 | |  | 377 | 415 | 908.4337349 | |  | 2066475 | 1836866.667 | 1877255.602 | |
|  |  | 50-59 | 18 | 19 | | 947.3684211 | |  | 614 | 684 | 897.6608187 | |  | 2148050 | 2034994.737 | 1928220.322 | |
|  |  | 60-69 | 5 | 5 | | 1000 | |  | 932 | 983 | 948.1180061 | |  | 1409745 | 1409745 | 1336604.619 | |
|  |  | 70-79 | 5 | 5 | | 1000 | |  | 1311 | 1366 | 959.7364568 | |  | 429235 | 429235 | 411952.478 | |
|  |  | >=80 | 3 | 3 | | 1000 | |  | 2588 | 2637 | 981.4182783 | |  | 323365 | 323365 | 317356.3216 | |
|  |  | Total | 314 | 344 | | 912.7906977 | |  | 17305 | 18593 | 930.7266175 | |  | 22001165 | 20587464.63 | 19544478.4 | |
|  |  |  |  |  | |  | |  |  |  |  | |  |  | DROHCA | COHCA | |
|  | |  |  | |  | |  |  |  | | | Adjusted Rates: | | | 935.7442951 | 888.3383404 |  |
