# Supplementary material for: Out-of-hospital cardiac arrest survival in drug-related versus cardiac causes in Ontario: A retrospective cohort study
Source: PLoS One. 2017 Apr 26;12(4):e0176441. doi: 10.1371/journal.pone.0176441 (PMC5405992; doi:10.1371/journal.pone.0176441)
Supplement: S3 Appendix — (DOCX) [file pone.0176441.s003.docx]

***Appendix 3: Sensitivity Analysis of Drug-Related Cardiac Arrest Case Definition***

| **Table 2a: Descriptive Analysis — Cardiac Arrest with Drug-Related and Presumed Cardiac Causes; Drug-Related Cardiac Arrest redefined as any cardiac arrest where the patient had a past history of drug use.** | | | |
| --- | --- | --- | --- |
|  | Drug-Related | Presumed Cardiac | p-value |
| **Patient Characteristics** | | | |
| Age |  |  |  |
| Mean, yr[95% CI] | 50.26 [49.28-51.23] | 68.93 [68.69-69.17] | **<0.0001** |
| ≤19, n(%) | 5 (0.49) | 390 (1.90) | **<0.0001** |
| 20-29, n(%) | 105 (10.39) | 234 (1.14) |  |
| 30-39, n(%) | 151 (14.94) | 485 (2.37) |  |
| 40-49, n(%) | 223 (22.06) | 1397 (2825) |  |
| 50-59, n(%) | 264 (26.11) | 2825 (13.79) |  |
| 60-69, n(%) | 148 (14.64) | 3855 (18.82) |  |
| 70-79, n(%) | 76 (7.52) | 4696 (22.92) |  |
| ≥80, n(%) | 39 (3.86) | 6604 (32.24) |  |
| Female Sex, n(%) | 281/1011 (27.79) | 7326/20486 (35.76)♮ | **<0.0001** |
| Public Location n(%) | 102/1011 (10.09) | 3245/20486 (15.87) | **<0.0001** |
| **Response** | | | |
| Bystander resuscitation*, n(%) | 351/918(38.2) | 7241/18021 (40.2) | 0.24 |
| Witnessed (Bystander or EMS), n(%) | 314/1011 (31.1) | 10678/20486 (52.1) | **<0.0001** |
| EMS Response Time*, minutes[95%CI]*(*n*)* | 6.31 [5.94-6.67] (836) | 6.40 [6.35-6.44] (16486) | 0.76 |
| ALS Response, n(%) | 940/1011 (93.0) | 18428/20486 (90.01) | **0.001** |
| Transported to ED, n(%) | 463/1011 (45.80) | 11691/20486 (57.07) | **<0.0001** |
| **Prehospital Resuscitation Procedure** | | | |
| Shockable Cardiac Rhythm, n(%)** | 93/973 (9.56) | 4725/19924 | **<0.0001** |
| Any prehospital Defibrillation, n(%) | 228/1011 (22.6) | 7204/20486 (35.2) | **<0.0001** |
| Time of call to first shock ♮♮, minutes[95% CI] | 12.49 (10.82-14.15] | 12.81[12.58-13.03] | 0.70 |
| Epinephrine, n(%) | 758/1011 (75.0) | 14800/20486 (72.2) | 0.06 |
| Amiodarone, n(%) | 50/1011 (4.95) | 1913/20486 (9.34) | **<0.0001** |
| Airway - Advanced, n(%) | 801/1011 (79.23) | 15496/20486 (75.64) | **0.01** |
| Compressions/min, mean[95% CI] | 108.55(107.45-109.65) | 108.77(108.52-109.02) | 0.62 |
| Comp’n Depth, cm, mean [95% CI] | 4.63 (4.27-4.99) | 4.59 (4.57-4.61) | 0.49 |
| **Outcome Variables** | | | |
| Discharge from Hospital | 66/1011 (6.53) | 1731/20486 (8.45) | **0.03** |
| ROSC ≥20 minutes after ED arrival §§, n(%) | 185/1009 (39.96) | 4433/20381 (21.75) | **0.01** |
| Admission to Hospital♮, n(%) | 198/1011 (19.58) | 4070/20486 (19.87) | 0.83 |
| Favourable Neurological Outcome (MRS ≤2)§, n(%) | 31/546 (5.68) | 828/9575 (8.65) | **0.03** |
| ALS: Advanced Life Support; AED: Automated External Defibrillator; CPR: Cardiopulmonary resuscitation; MRS: Modified Rankin Score; EMS: Emergency Medical Services; ROSC: Return of Spontaneous Circulation; ED: Emergency Department  *EMS-witnessed events excluded; ♮♮Among Shockable patients, n=4344; § for cases after Jan 1, 2011, 21 missing values in the presumed cardiac group; ♮ 2 missing values; **comparison made without missing or unknown values; §§ 106 missing values, 2 in the Drug-Related Cardiac Arrest group | | | |

| **Table 3a Logistic regression sensitivity analysis**  **Effect of Drug-Related vs. Presumed Cardiac Cause on Survival to Hospital Discharge** | | | | | | |
| --- | --- | --- | --- | --- | --- | --- |
|  | **Primary Analysis** | | **Sensitivity Analysis A:**  Cases with Complete Data | | **Sensitivity Analysis B:**  Drug-Related Cardiac Arrest redefined as any Out-of-Hospital Cardiac Arrest where patient had a past history of drug use | |
| Variable/Covariate | OR | 95% CI | OR | 95% CI | OR | 95% CI |
| Drug-Related vs. Presumed Cardiac (ref.) | 1.44 | 1.15-1.81 | 2.25 | 1.41-3.60 | 1.11 | 0.93-1.32 |
| Age |  |  |  |  |  |  |
| ≤19 | 1.32 | 0.93-1.89 | 1.35 | 0.87-2.09 | 1.30 | 0.91-1.86 |
| 20-29 | 2.15 | 1.48-3.12 | 2.00 | 1.25-3.18 | 2.23 | 1.53-3.24 |
| 30-39 | 2.07 | 1.57-2.73 | 1.90 | 1.32-2.71 | 2.17 | 1.65-2.86 |
| 40-49 | 1.09 | 0.89-1.33 | 1.15 | 0.90-1.50 | 1.08 | 0.89-1.33 |
| 50-59 (ref.) | - | - | - | - | - | - |
| 60-69 | 0.86 | 0.74-1.01 | 0.88 | 0.71-1.08 | 0.85 | 0.73-0.99 |
| 70-79 | 0.60 | 0.51.0.71 | 0.62 | 0.49-0.77 | 0.59 | 0.50-0.69 |
| ≥80 | 0.30 | 0.25-0.36 | 0.33 | 0.26-0.42 | 0.30 | 0.24-0.35 |
| EMS response time (per minute) | 0.91 | 0.89-0.95 | 0.90 | 0.88-0.94 | 0.92 | 0.89-0.95 |
| ALS response (vs. BLS) | 2.65 | 2.33-3.02 | 6.59 | 4.95-8.77 | 2.66 | 2.33-3.02 |
| Witnessed OHCA | 1.78 | 1.64-1.93 | 3.07 | 2.58-3.64 | 0.31 | 0.29-0.34 |
| Epinephrine given | 0.31 | 0.28-0.34 | 0.10 | 0.09-0.12 | 1.75 | 1.62-1.91 |
| Advanced airway inserted | 0.80 | 0.73-0.86 | 0.62 | 0.52-0.73 | 0.80 | 0.74-0.87 |
| VF or VT (vs. other rhythm) | 3.16 | 2.91-3.43 | 10.08 | 8.48-11.97 | 3.15 | 2.90-3.42 |
| Bystander resuscitation | 1.11 | 1.04-1.20 | 1.27 | 1.10 -1.48 | 1.11 | 1.03-1.19 |
| Public vs. private location | 1.27 | 1.18-1.37 | 1.59 | 1.36-1.86 | 1.27 | 1.18-1.37 |
| Sensitivity Analysis A Model fit statistics: R^2^=0.1749, Adjusted R^2^=0.4398, c=0.905  Sensitivity Analysis B Model fit statistics: R^2^=0.1741, Adjusted R^2^=0.4390, c=0.905  ALS: Advanced Life Support, OHCA: Out-of-hospital cardiac arrest, VF: Ventricular Fibrillation, VT: Ventricular Tachycardia | | | | | | |
